# Intestinal and Systemic Immune Responses upon Multi-drug Resistant *Pseudomonas aeruginosa* Colonization of Mice Harboring a Human Gut Microbiota

**DOI:** 10.3389/fmicb.2017.02590

**Published:** 2017-12-22

**Authors:** Eliane von Klitzing, Ira Ekmekciu, Stefan Bereswill, Markus M. Heimesaat

**Affiliations:** Institute of Microbiology, Charité – Universitätsmedizin Berlin, Corporate Member of Freie Universität Berlin, Humboldt-Universität zu Berlin, and Berlin Institute of Health, Department of Microbiology and Hygiene, Berlin, Germany

**Keywords:** multidrug-resistant Gram-negative bacteria, intestinal *Pseudomonas aeruginosa* colonization, fecal microbiota transplantation, human microbiota associated mice, colonization resistance, host-pathogen-interaction, intestinal and systemic sequelae of colonization, broad-spectrum antibiotic treatment

## Abstract

The World Health Organization has rated multi-drug resistant (MDR) *Pseudomonas aeruginosa* as serious threat for human health. It is, however, unclear, whether intestinal MDR *P. aeruginosa* carriage is associated with inflammatory responses in intestinal or even systemic compartments. In the present study, we generated with respect to their microbiota “humanized” mice by human fecal microbiota transplantation of secondary abiotic mice. Following peroral challenge with a clinical *P. aeruginosa* isolate on two consecutive days, mice harboring a human or murine microbiota were only partially protected from stable intestinal *P. aeruginosa* colonization given that up to 78% of mice were *P. aeruginosa*-positive at day 28 post-infection (p.i.). Irrespective of the host-specificity of the microbiota, *P. aeruginosa* colonized mice were clinically uncompromised. However, *P. aeruginosa* colonization resulted in increased intestinal epithelial apoptosis that was accompanied by pronounced proliferative/regenerative cell responses. Furthermore, at day 7 p.i. increased innate immune cell populations such as macrophages and monocytes could be observed in the colon of mice harboring either a human or murine microbiota, whereas this held true at day 28 p.i. for adaptive immune cells such as B lymphocytes in both the small and large intestines of mice with murine microbiota. At day 7 p.i., pro-inflammatory cytokine secretion was enhanced in the colon and mesenteric lymph nodes, whereas the anti-inflammatory cytokine IL-10 was down-regulated in the former at day 28 p.i. Strikingly, cytokine responses upon intestinal *P. aeruginosa* colonization were not restricted to the intestinal tract, but could also be observed systemically, given that TNF and IFN-γ concentrations were elevated in spleens as early as 7 days p.i., whereas splenic IL-10 levels were dampened at day 28 p.i. of mice with human microbiota. In conclusion, mere intestinal carriage of MDR *P. aeruginosa* by clinically unaffected mice results in pro-inflammatory sequelae not only in intestinal, but also systemic compartments.

## Introduction

*Pseudomonas aeruginosa* constitute strictly aerobic Gram-negative bacteria that normally inhabit the soil and surfaces in aqueous environments. Their adaptive capabilities and intrinsic antibiotic resistance support survival in nature as well as in a plethora of settings including healthcare facilities (Gellatly and Hancock, 2013). Whereas a single flagellum and many cell surface pili facilitate bacterial motility and adherence to surfaces (Driscoll et al., 2007), alginate secretion, biofilm formation, quorum-sensing and an elaborate secretion system contribute to *P. aeruginosa* virulence (Driscoll et al., 2007; Gellatly and Hancock, 2013). In the hospital, typical environmental reservoirs of *P. aeruginosa* include sinks, water bottles and respiratory equipment, for instance (Morrison and Wenzel, 1984; Stratton, 1990). *P. aeruginosa* is a leading cause of nosocomial infections in ICUs such as ventilator-associated pneumonia, bloodstream infections, urinary tract infections or superinfections of burn wounds associated with mortality rates of more than 30% (Vincent, 2003). The emergence of MDR *P. aeruginosa* strains particularly due to extended-spectrum β-lactamases, carbapenemases and 16S rRNA methylases, has posed immuno-compromised individuals, patients suffering from cystic fibrosis and other chronic pulmonary morbidities or ICU patients at increased risk for prolonged hospital stay and mortality (Vincent, 2003; Oliver et al., 2015; Potron et al., 2015). In consequence, the WHO has rated MDR Gram-negative bacteria including *P. aeruginosa* as serious threat for human health and emphasized the urgent need for new treatment options (Tacconelli and Magrini, 2017). *P. aeruginosa* infection typically follows colonization that may happen either endogenously (hence arising from the patients’ inherent microbiota) or exogenously (if acquired from other patients or from the hospital environment by cross-contamination) (Cohen et al., 2017). Intestinal *P. aeruginosa* colonization before ICU admission has been shown to be associated with an almost 15 times increased risk of subsequent infection as compared to non-colonized individuals (Gomez-Zorrilla et al., 2015). Disruption of the complex and diverse commensal intestinal microbiota physiologically exerting colonization resistance to the host by antimicrobial compounds further facilitates establishment and invasion of opportunistic or obligate pathogens in mice and men (Hentges et al., 1985; Marshall et al., 1993; Bereswill et al., 2011; Fiebiger et al., 2016). We have recently shown that also the host-specificity of the complex intestinal microbiota is pivotal for effective colonization resistance given that mice harboring a murine, but not human gut microbiota were protected from intestinal colonization with enteropathogenic *C. jejuni* (Bereswill et al., 2011). Conversely, perorally with *C. jejuni* challenged “humanized” mice could be stably infected by the enteropathogen and displayed pro-inflammatory immune responses as seen in human campylobacteriosis (Bereswill et al., 2011; Masanta et al., 2013; Fiebiger et al., 2016).

To date, however, no data are available regarding host immune responses in otherwise healthy *P. aeruginosa* carriers with an intact intestinal microbiota composition. In the present study we therefore investigated, whether mice harboring a human vs. murine microbiota could be stably colonized by a clinical MDR *P. aeruginosa* strain and whether bacterial carriage was associated with intestinal or even systemic inflammatory host responses.

## Materials and Methods

### Ethics Statement

All animal experiments were conducted according to the European Guidelines for animal welfare (2010/63/EU) with approval of the commission for animal experiments headed by the “Landesamt für Gesundheit und Soziales” (LaGeSo, Berlin; registration numbers G0039/15). Animal welfare was monitored twice daily by assessment of clinical conditions and weight loss of mice.

### Generation of Secondary Abiotic Mice

Female C57BL/6j mice were bred and maintained under SPF conditions in the Forschungsinstitute für Experimentelle Medizin (Charité – University Medicine, Berlin, Germany). Secondary abiotic mice with a virtually depleted microbiota were generated as described previously (Heimesaat et al., 2006). In brief, 8 weeks old mice were transferred into sterile cages and subjected to a broad-spectrum antibiotic treatment for 8 weeks by adding ampicillin plus sulbactam (1 g/L; Ratiopharm, Germany), vancomycin (500 mg/L; Cell Pharm, Germany), ciprofloxacin (200 mg/L; Bayer Vital, Germany), imipenem (250 mg/L; MSD, Germany) and metronidazole (1 g/L; Fresenius, Germany) to the drinking water (*ad libitum*). Cultural and culture-independent (i.e., 16S rRNA based molecular) quality control measures revealed virtual absence of bacteria in fecal samples as described earlier (Ekmekciu et al., 2017).

### Human and Murine Fecal Microbiota Transplantation

Three days before association of secondary abiotic mice with a complex human or murine intestinal microbiota by FMT, the antibiotic cocktail was replaced by autoclaved tap water (*ad libitum*). Fresh fecal samples free of enteropathogenic bacteria (e.g., enteropathogenic *Escherichia coli*, *Salmonella*, *Shigella*, *Yersinia*, *Campylobacter*, *Clostridium difficile*), viruses (e.g., Noro-, Rota-, Enterovirus) and parasites (e.g., *Giardia lamblia*, *Entamoeba histolytica*, *Blastocystis hominis* and worms) were collected from five individual healthy human volunteers, dissolved in sterile PBS (Gibco, Life Technologies, United Kingdom), aliquoted and stored at -80°C as described earlier (Bereswill et al., 2011). Immediately before FMT, individual fecal aliquots were thawed and pooled (Bereswill et al., 2011). In addition, fresh murine fecal samples were collected from 10 age and sex matched SPF control mice, pooled, dissolved in 10 mL sterile PBS and the supernatant served as murine donor suspension. To generate human intestinal microbiota associated mice or reintroduce murine microbiota into mice, secondary abiotic animals were subjected to peroral FMT either with 0.3 mL of the human or murine donor suspension by gavage on two consecutive days (Bereswill et al., 2011). Of note, each donor suspension was tested negative for *P. aeruginosa* by both culture and culture-independent (i.e., 16S rRNA based) methods. Bacterial groups varied less than 0.5 logarithmic orders of magnitude between independent experiments. To assure proper establishment of the human or murine microbiota in the murine host, mice were kept for 3 weeks until *P. aeruginosa* infection. Immediately before peroral *P. aeruginosa* challenge individual fecal samples were collected for quantitative molecular analyses of main intestinal bacterial communities as described elsewhere (Bereswill et al., 2011).

### Molecular Analysis of the Human and Murine Donor Suspensions and the Fecal Microbiota

DNA was extracted from fecal samples as described previously (Heimesaat et al., 2006). In brief, DNA was quantified by using Quant-iT PicoGreen reagent (Invitrogen, United Kingdom) and adjusted to 1 ng per μL. Then, TLs as well as the main bacterial groups abundant in the murine and human intestinal microbiota including EB, EC, LB, Bif, B/P, Clocc, Clept and MIB were assessed by qRT-PCR with species-, genera- or group-specific 16S rRNA gene primers (Tib MolBiol, Germany) as described previously (Heimesaat et al., 2010) and numbers of 16S rRNA gene copies per ng DNA of each sample determined.

### MDR *P. aeruginosa* Infection and Quantitative Assessment of Fecal Loads

The MDR *P. aeruginosa* isolate was initially cultured from respiratory material of a patient suffering from nosocomial pneumonia and kindly provided by Prof. Dr. Bastian Opitz (Charité – University Medicine, Berlin, Germany). Notably, the clinical bacterial strain was tested resistant against piperacillin/tazobactam, ceftazidime, cefepim ± cefoxitin, imipenem, ciprofloxacin, gentamycin, tobramycin, amikacin, trimethoprim/sulfamethoxazole, and aztreonam (according to EUCAST interpretation guidelines) and displayed antimicrobial sensitivity to fosfomycin and colistin only (von Klitzing et al., 2017a). Prior infection, the *P. aeruginosa* strain was grown on cetrimide agar (Oxoid) for 48 h in an aerobic atmosphere at 37°C.

On days 0 and 1, mice were perorally challenged with 10^9^ CFU of the MDR *P. aeruginosa* strain by gavage in a total volume of 0.3 mL PBS as reported earlier (von Klitzing et al., 2017a).

For quantitative assessment of fecal *P. aeruginosa* loads over time p.i., fecal samples were homogenized in sterile PBS, then serial dilutions streaked onto Columbia agar supplemented with 5% sheep blood (Oxoid, Germany) and cetrimide agar and incubated in an aerobic atmosphere at 37°C for 48 h as described previously (von Klitzing et al., 2017a). Fecal weights were determined by the difference of the sample weights before and after asservation. The detection limit of viable bacteria was 100 CFU per g.

### Clinical Conditions

Macroscopic and/or microscopic abundance of fecal blood was assessed in individual mice on a daily basis by the Guajac method using Haemoccult (Beckman Coulter/ PCD, Germany) as described earlier (Haag et al., 2012a).

### Sampling Procedures

Mice were sacrificed 28 days p.i. by isoflurane treatment (Abott, Germany). Cardiac blood (for serum) and tissue samples from spleen, MLN, ileum and colon were removed under sterile conditions. Intestinal samples were collected from each mouse in parallel for microbiological, immunological and immunohistochemical analyses.

### Immunohistochemistry

Five μm thin paraffin sections of colonic and ileal *ex vivo* biopsies were used for *in situ* immunohistochemical analysis as reported previously (Heimesaat et al., 2010; Alutis et al., 2015a,b). In brief, primary antibodies against cleaved caspase-3 (Asp175, Cell Signaling, Beverly, MA, United States, 1:200), Ki67 (TEC3, Dako, Glostrup, Denmark, 1:100), F4/80 (#14-4801, clone BM8, eBioscience, 1:50) and B220 (eBioscience, 1:200) were used to assess apoptotic cells, proliferating cells, macrophages/monocytes, and B lymphocytes, respectively. The average numbers of positively stained cells within at least six HPFs (0.287 mm^2^; 400× magnification) were determined by an independent blinded investigator.

### Cytokine Detection

*Ex vivo* biopsies (approximately 1 cm^2^) derived from colon and ileum (both cut longitudinally and washed in PBS), as well as MLN and spleen were placed into 24-flat-bottom well culture plates (Falcon, Germany) containing 500 mL serum-free RPMI 1640 medium (Gibco, Life Technologies) supplemented with penicillin (100 U/ml, Biochrom, Germany) and streptomycin (100 μg/ml; Biochrom). After 18 h at 37°C, culture supernatants and serum samples were tested for TNF, IFN-γ and IL-10 by the Mouse Inflammation Cytometric Bead Assay (CBA; BD Bioscience) on a BD FACSCanto II flow cytometer (BD Bioscience) as described earlier (Haag et al., 2012b).

### Statistical Analysis

Medians and levels of significance were determined using appropriate tests (Mann–Whitney *U* test, one-way ANOVA and Kruskal–Wallis test) as indicated. Two-sided probability (*p*) values ≤ 0.05 were considered significant. Experiments were reproduced at least twice.

## Results

### Intestinal *P. aeruginosa* Colonization in Mice Harboring a Complex Human vs. Murine Gut Microbiota

**FIGURE 1:**
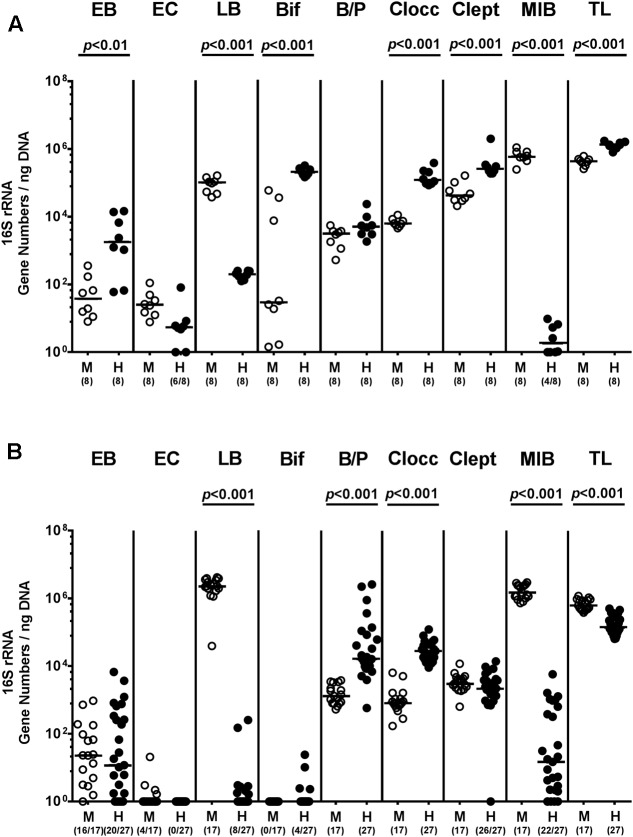
Intestinal bacterial colonization following human and murine FMT of secondary abiotic mice. Secondary abiotic mice were generated by broad-spectrum antibiotic treatment and associated with either a complex murine (M, open circles) or human (H, closed circles) intestinal microbiota by FMT on two consecutive days. **(A)** Respective donor suspensions and **(B)** individual fecal sample taken three weeks following FMT (and hence, immediately before MDR *P. aeruginosa* challenge) were subjected to a comprehensive fecal microbiota survey by quantitative Real-Time PCR amplifying variable regions of the bacterial 16S rRNA gene. The following main intestinal bacterial groups were determined (expressed as 16S rRNA gene numbers per ng DNA): Enterobacteria (EB), enterococci (EC), lactic acid bacteria (LB), bifidobacteria (Bif), *Bacteroides/Prevotella* spp. (B/P), *Clostridium coccoides* group (Clocc), *Clostridium leptum* group (Clept), *Mouse Intestinal Bacteroides* (MIB), and total eubacterial load (TL). Medians (black bars) and significance levels (*p*-values) determined by Mann–Whitney *U*-test are shown. Numbers of samples harboring the respective bacterial group out of the total number of analyzed samples are given in parentheses. Data were pooled from four independent experiments.

In the present study we addressed whether a complex human or murine gut microbiota could sufficiently confer colonization resistance against MDR *P. aeruginosa* to the vertebrate host and if not, whether intestinal *P. aeruginosa* carriage resulted in intestinal or even systemic inflammatory sequelae. To accomplish this, secondary abiotic mice were associated with either a human or murine microbiota by peroral FMT. Culture-independent (i.e., molecular) analyses of the microbiota suspensions derived from human or murine donors revealed substantial host-specific differences in bacterial compositions. TLs and those of specific families/genera such as EB, Bif, Clocc, and Clept were higher in human as compared to murine bacterial suspensions (*p* < 0.01–0.001; **Figure 1A**), whereas LB or MIB were lower or even virtually absent in the former as compared to the latter (*p* < 0.001; **Figure 1A**). Three weeks following FMT and immediately before *P. aeruginosa* challenge (i.e., day 0), we further assessed respective microbiota compositions in fecal samples derived from individual mice. Molecular analyses revealed higher fecal gene numbers of B/P and Clept, but lower LB and MIB burdens as well as slightly lower total eubacterial gene numbers (less than one order of magnitude) in human microbiota transplanted mice as compared to murine counterparts (*p* < 0.001; **Figure 1B**). These results thus indicate stable intestinal establishment of distinct microbiota following respective FMT.

**FIGURE 2:**
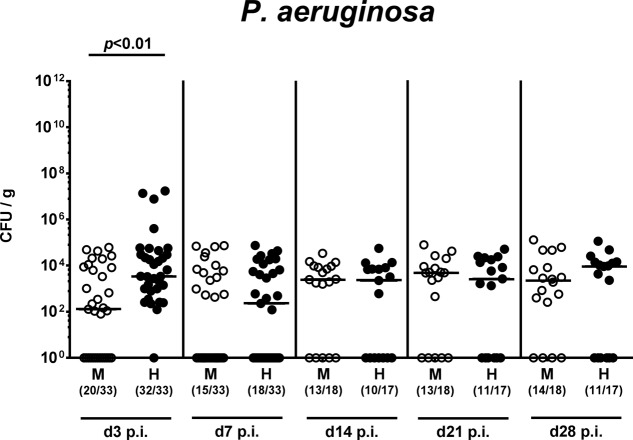
Intestinal colonization densities of MDR *P. aeruginosa* in mice harboring a human vs. murine microbiota. Mice with a murine (M, open circles) or human (H, closed circles) intestinal microbiota were perorally challenged with MDR *P. aeruginosa* on day 0 and day 1. Intestinal colonization densities were assessed in fecal samples at defined time points p.i. by culture. Medians (black bars) and significance levels (*p*-values) determined by Mann–Whitney *U*-test are shown. Numbers of samples harboring the respective bacterial group out of the total number of analyzed samples are given in parentheses. Data were pooled from four independent experiments.

On two consecutive days (i.e., days 0 and 1), we perorally challenged mice with 10^9^ viable *P. aeruginosa* by gavage and quantitatively assessed fecal *P. aeruginosa* loads thereafter. Cultural analyses revealed that as early as 48 h following the latest bacterial challenge (i.e., day 3 p.i.), virtually all human microbiota transplanted mice harbored *P. aeruginosa* in their intestines with median loads of approximately 10^4^ CFU per g, whereas bacterial counts were approximately two orders of magnitude lower in murine controls (*p* < 0.01; **Figure 2** and **Supplementary Figure S1**). Of note, by day 3 p.i. *P. aeruginosa* could not be detected in 40% of mice with a murine gut microbiota. Until the end of the observation period, however, fecal *P. aeruginosa* loads were comparable in mice with either microbiota, whereas 64.7% of mice following human FMT and 77.8% of murine controls harbored the bacterial strain in their intestinal tract at day 28 p.i. (**Figure 2** and **Supplementary Figure S1**). Hence, the complex intestinal microbiota, irrespective whether of human or murine origin, prevented only a subgroup of mice from long-term intestinal MDR *P. aeruginosa* colonization.

### Intestinal Microbiota Changes upon *P. aeruginosa* Colonization of Mice Harboring a Complex Human vs. Murine Gut Microbiota

**FIGURE 3:**
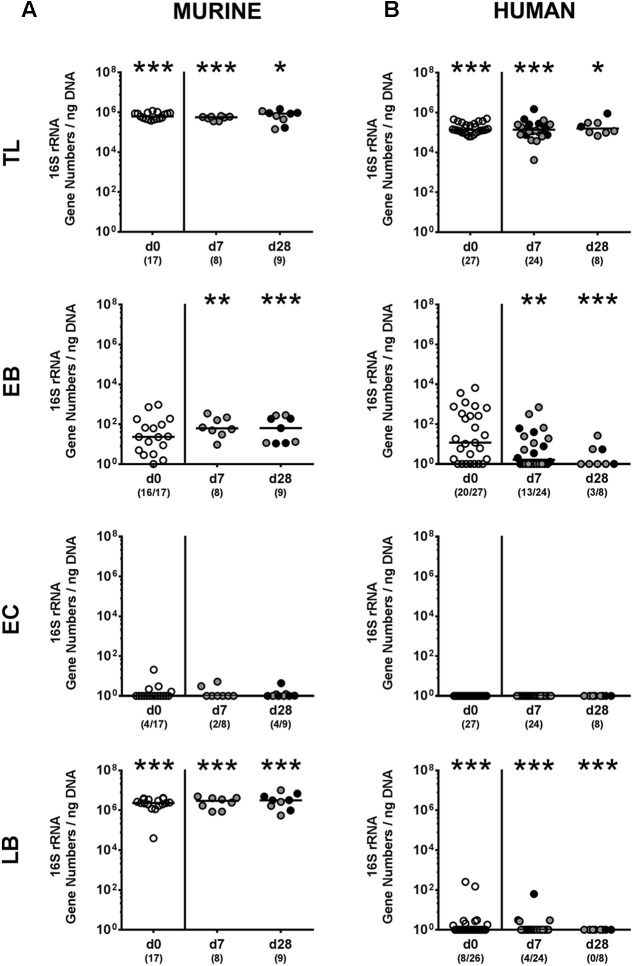
Intestinal total and aerobic bacterial changes following MDR *P. aeruginosa* colonization of mice harboring a human vs. murine microbiota. Mice with a **(A)** murine or **(B)** human intestinal microbiota were perorally challenged with *P. aeruginosa* on day 0 and day 1. Immediately before (day 0, open circles) and at day 7 and day 28 post-challenge the intestinal microbiota composition of colonic *P. aeruginosa* carrying (black circles) and non-carrying (gray circles) mice was surveyed applying quantitative Real-Time PCR amplifying variable regions of the bacterial 16S rRNA gene. The following main intestinal bacterial groups were determined (expressed as 16S rRNA gene numbers per ng DNA): Total eubacterial load (TL), enterobacteria (EB), enterococci (EC), and lactic acid bacteria (LB). Medians (black bars) and significance levels (*p*-values) determined by one-way ANOVA or Kruskal–Wallis test (according to data distribution) are shown. Significant differences between mice with a murine vs. human microbiota at defined time points are indicated by asterisks (^∗^*p* < 0.05; ^∗∗^*p* < 0.01; ^∗∗∗^*p* < 0.001), whereas numbered *p*-values indicate differences between mice harboring the same microbiota. Numbers of samples harboring the respective bacterial group out of the total number of analyzed samples are given in parentheses. Data were pooled from four independent experiments.

**FIGURE 4:**
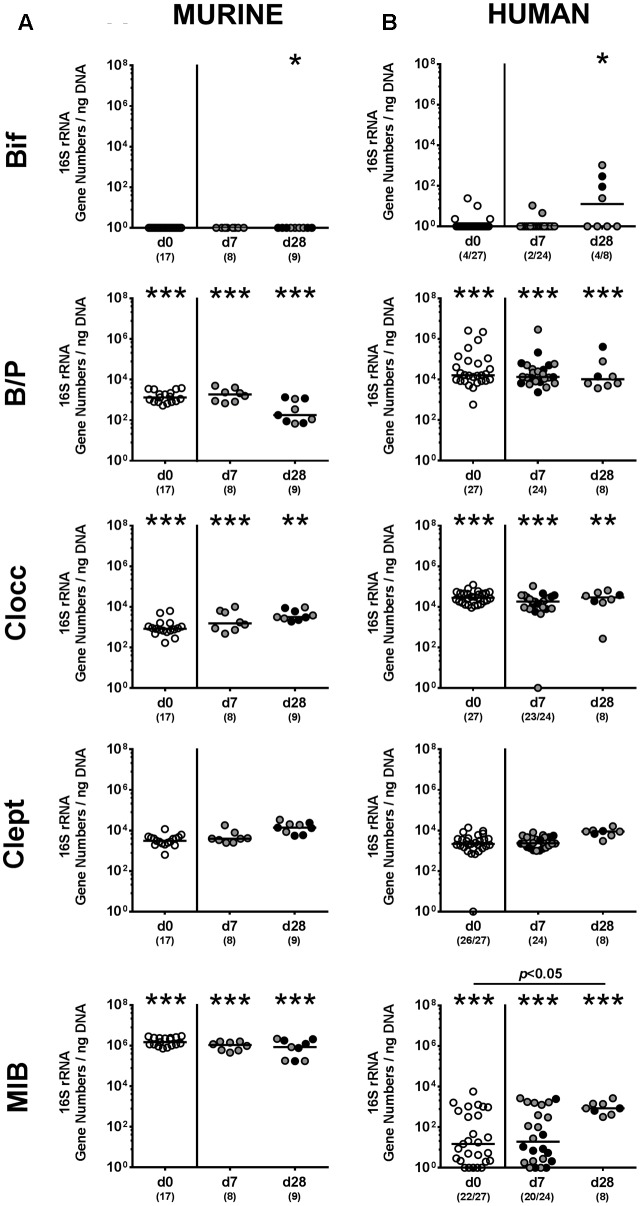
Intestinal anaerobic bacterial changes following MDR *P. aeruginosa* colonization of mice harboring a human vs. murine microbiota. Mice with a **(A)** murine or **(B)** human intestinal microbiota were perorally challenged with *P. aeruginosa* on day 0 and day 1. Immediately before (day 0, open circles) and at day 7 and day 28 post-challenge the intestinal anaerobic microbiota composition of colonic *P. aeruginosa* carrying (black circles) and non-carrying (gray circles) mice was surveyed applying quantitative Real-Time PCR amplifying variable regions of the bacterial 16S rRNA gene. The following main intestinal bacterial groups were determined (expressed as 16S rRNA gene numbers per ng DNA): Bifidobacteria (Bif), *Bacteroides/Prevotella* spp. (B/P), *Clostridium coccoides* group (Clocc), *Clostridium leptum* group (Clept), and *Mouse Intestinal Bacteroides* (MIB). Medians (black bars) and significance levels (*p*-values) determined by one-way ANOVA or Kruskal–Wallis test (according to data distribution) are shown. Significant differences between mice with a murine vs. human microbiota at defined time points are indicated by asterisks (^∗^*p* < 0.05; ^∗∗^*p* < 0.01; ^∗∗∗^*p* < 0.001), whereas numbered *p*-values indicate differences between mice harboring the same microbiota. Data were pooled from four independent experiments.

We next surveyed whether *P. aeruginosa* colonization was accompanied by distinct changes of the intestinal microbiota composition over time (**Figures 3**, **4**). Quantitative culture-independent analysis revealed that four weeks following *P. aeruginosa* association, intestinal gene numbers of MIB had increased in mice that had been subjected to human FMT, but not conventionally colonized mice (*p* < 0.05; **Figure 3**). As early as one week following *P. aeruginosa* challenge, however, the respective gut microbiota did not differ from uninfected control groups, neither in mice harboring a human, nor murine microbiota (**Figures 3**, **4**). Hence, no overt changes in intestinal microbiota composition could be observed relatively early (i.e., 1 week) upon *P. aeruginosa* colonization and were rather subtle thereafter.

### Apoptotic and Proliferating Responses in Intestinal Epithelial Cells upon *P. aeruginosa* Colonization of Mice Harboring a Complex Human vs. Murine Gut Microbiota

**FIGURE 5:**
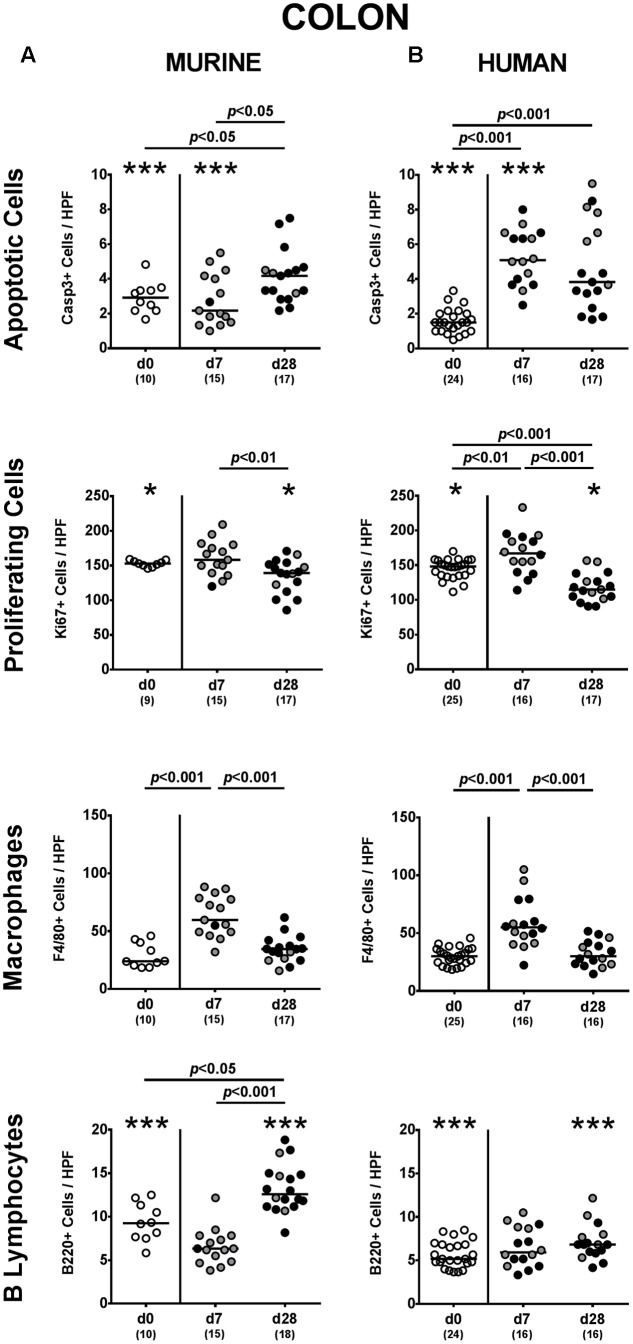
Apoptotic and proliferating epithelial cell as well as immune cell responses in the large intestines following MDR *P. aeruginosa* colonization of mice harboring a human vs. murine microbiota. Mice with a **(A)** murine or **(B)** human intestinal microbiota were perorally challenged with MDR *P. aeruginosa* on day 0 and day 1. At day 7 and day 28 post-challenge, the average number of apoptotic (Casp3+) and proliferating (Ki67+) cells as well as of macrophages/monocytes (F4/80+) and B lymphocytes (B220+) in at least six HPF were quantitatively assessed in colonic paraffin sections derived from colonic *P. aeruginosa* carrying (black circles) and non-carrying (gray circles) mice applying *in situ* immunohistochemistry. Unchallenged mice (day 0, open circles) harboring a respective murine or human gut microbiota served as negative controls. Medians (black bars) and significance levels (*p*-values) determined by one-way ANOVA or Kruskal–Wallis test (according to data distribution) are shown. Significant differences between mice with a murine vs. human microbiota at defined time points are indicated by asterisks (^∗^*p* < 0.05; ^∗∗∗^*p* < 0.001), whereas numbered *p*-values indicate differences between mice harboring the same microbiota. Numbers of analyzed samples are given in parentheses. Data were pooled from four independent experiments.

Given that mice were not clinically compromised following *P. aeruginosa* challenge and did not display symptoms such as weight loss, diarrhea or fecal blood (data not shown), we next assessed potential microscopic sequelae of *P. aeruginosa* colonization. To address this, we quantitatively determined apoptotic epithelial cells in the large intestines applying *in situ* immunohistochemistry. Mice following human FMT displayed more than two-fold increased caspase-3 positive apoptotic cell numbers in their colonic epithelia at both day 7 and day 28 p.i. (*p* < 0.001; **Figure 5B**), whereas this was only the case later in the course of colonization of murine controls (*p* < 0.05; **Figure 5A**). Of note, at day 7 p.i. apoptotic epithelial cells were higher in the large intestines of mice that had been challenged with human gut microbiota as compared to murine controls (*p* < 0.001; **Figures 5A,B**). In the small intestines of mice with either microbiota, however, apoptotic cells were slightly higher at day 7 p.i. as compared to non-challenged controls (*p* < 0.05–0.01; **Supplementary Figures S2A,B**) and further increased until the end of the observation period (*p* < 0.05–0.001 vs. day 7 p.i.; **Supplementary Figures S2A,B**).

We additionally assessed Ki67 positive cell numbers in intestinal epithelia indicative for proliferative/regenerative cell responses counteracting potential *P. aeruginosa*-induced intestinal inflammatory sequelae. Remarkably, only in mice following human FMT, Ki67 positive cells were elevated in large intestines as early as 7 day p.i. (*p* < 0.01 vs. none-challenged controls), but declined thereafter (*p* < 0.001; **Figure 5B**). In the small intestines, proliferative cell responses were highest early in the course of *P. aeruginosa* colonization, whereas Ki67 positive ileal epithelial cell numbers declined thereafter (**Supplementary Figure S2**). Hence, *P. aeruginosa* colonization of mice resulted in increased intestinal epithelial apoptosis that was accompanied by proliferative/regenerative epithelial cell responses.

### Intestinal Immune Cell Responses upon *P. aeruginosa* Colonization of Mice Harboring a Complex Human vs. Murine Gut Microbiota

We next addressed whether colonization of human fecal microbiota transplanted mice with MDR *P. aeruginosa* was accompanied by an increased influx of distinct immune cell populations into the intestinal tract, again applying quantitative *in situ* immunohistochemistry. Irrespective of the host-specificity of the intestinal microbiota, numbers of innate immune cells such as F4/80 positive macrophages and monocytes were elevated in the large intestinal mucosa and lamina propria at day 7 p.i., but declined thereafter (*p* < 0.001; **Figure 5**). Only in mice with a murine microbiota, however, *P. aeruginosa*-induced adaptive immune cell responses were more pronounced later in the course of colonization as indicated by increased numbers of colonic B lymphocytes at day 28 p.i. (*p* < 0.05 vs. non-challenged controls; **Figure 5A**). In the small intestinal tract, increases in both numbers of macrophages/monocytes as well as of B lymphocytes could only be observed later in the course of *P. aruginosa* colonization in conventionally colonized, but not human fecal microbiota transplanted mice (*p* < 0.01–0.001; **Supplementary Figure S2A**). Hence, rather early in the course of *P. aeruginosa* colonization increased innate immune cell subsets could be observed in the large intestines of mice harboring either a human or murine gut microbiota, whereas this held true for adaptive immune cells such as B lymphocytes in the small and large intestinal tract of mice with a murine microbiota rather later in the course of *P. aeruginosa* colonization.

### Intestinal Cytokine Responses upon *P. aeruginosa* Colonization of Mice Harboring a Complex Human vs. Murine Gut Microbiota

**FIGURE 6:**
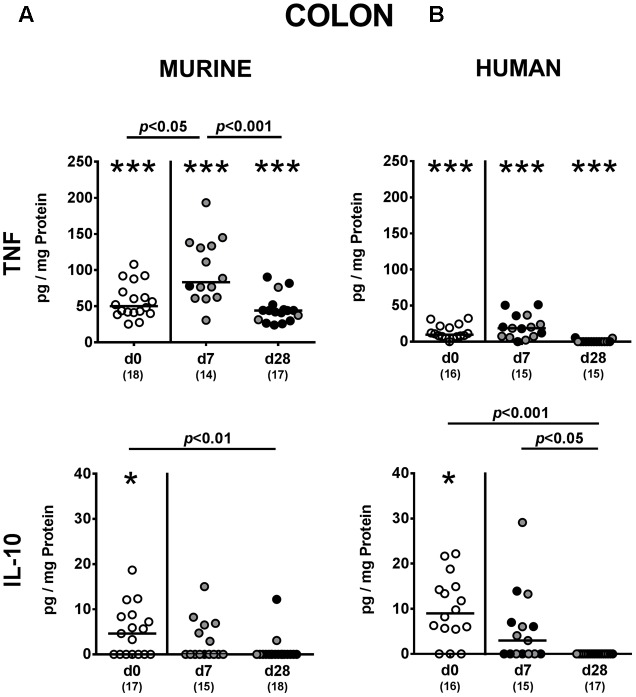
Colonic pro- and anti-inflammatory cytokine responses following MDR *P. aeruginosa* colonization of mice harboring a human vs. murine microbiota. Mice with a **(A)** murine or **(B)** human intestinal microbiota were perorally challenged with MDR *P. aeruginosa* on day 0 and day 1. At day 7 and day 28 post-challenge, TNF and IL-10 concentrations were determined in colonic *ex vivo* biopsies derived from colonic *P. aeruginosa* carrying (black circles) and non-carrying (gray circles) mice. Unchallenged mice (day 0, open circles) harboring a respective murine or human gut microbiota served as negative controls. Medians (black bars) and significance levels (*p*-values) determined by one-way ANOVA or Kruskal–Wallis test (according to data distribution) are shown. Significant differences between mice with a murine vs. human microbiota at defined time points are indicated by asterisks (^∗^*p* < 0.05; ^∗∗∗^*p* < 0.001), whereas numbered *p*-values indicate differences between mice harboring the same microbiota. Numbers of analyzed samples are given in parentheses. Data were pooled from four independent experiments.

**FIGURE 7:**
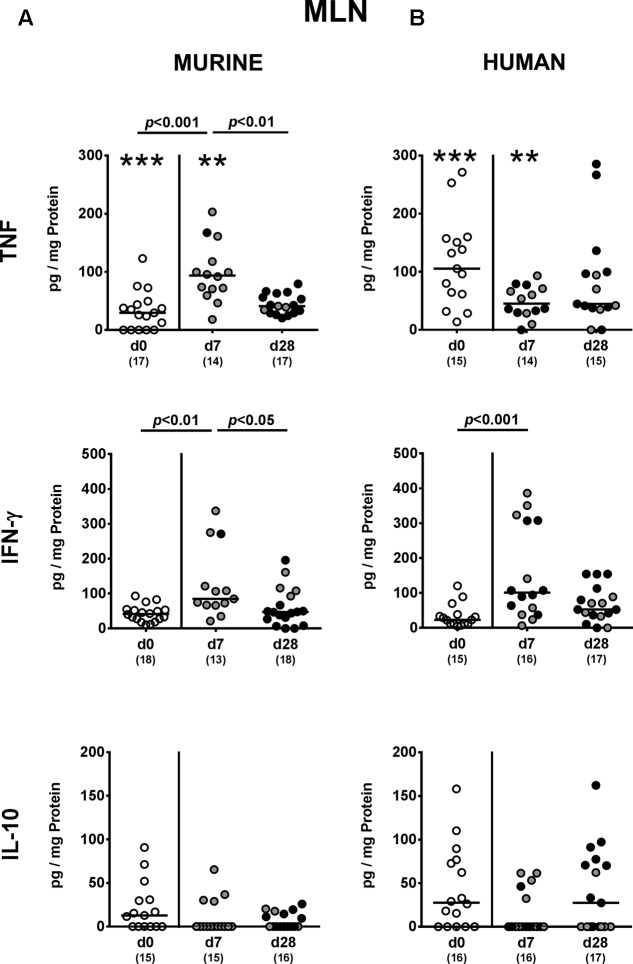
Pro- and anti-inflammatory cytokine responses in MLN following MDR *P. aeruginosa* colonization of mice harboring a human vs. murine microbiota. Mice with a **(A)** murine or **(B)** human intestinal microbiota were perorally challenged with MDR *P. aeruginosa* on day 0 and day 1. At day 7 and day 28 post-challenge, TNF, IFN-γ and IL-10 concentrations were determined in *ex vivo* biopsies derived from MLN of colonic *P. aeruginosa* carrying (black circles) and non-carrying (gray circles) mice. Unchallenged mice (day 0, open circles) harboring a respective murine or human gut microbiota served as negative controls. Medians (black bars) and significance levels (*p*-values) determined by one-way ANOVA or Kruskal–Wallis test (according to data distribution) are shown. Significant differences between mice with a murine vs. human microbiota at defined time points are indicated by asterisks (^∗∗^*p* < 0.01; ^∗∗∗^*p* < 0.001), whereas numbered *p*-values indicate differences between mice harboring the same microbiota. Numbers of analyzed samples are given in parentheses. Data were pooled from four independent experiments.

We next addressed whether changes in intestinal innate and adaptive immune cell numbers upon *P. aeruginosa* colonization of human vs. murine fecal microbiota transplanted mice was accompanied by pro- and/or anti-inflammatory cytokine responses within the intestinal tract. At day 7 p.i., increased concentrations of the pro-inflammatory cytokine TNF could be detected in colonic *ex vivo* biopsies derived from mice harboring a murine, but not human microbiota (*p* < 0.05; **Figure 6A**). Large intestinal secretion of the anti-inflammatory cytokine IL-10, however, was dampened at day 7 and day 28 p.i. of mice that had been subjected to human FMT (*p* < 0.001 and *p* < 0.05, respectively; **Figure 6B**) and at the later time point in mice with a murine microbiota (*p* < 0.01; **Figure 6A**). We further analyzed cytokine secretion in MLN. Alike the colon, TNF concentrations were elevated in MLN as early as 7 day p.i. of mice harboring a murine microbiota only (*p* < 0.001), but declined thereafter to basal levels (*p* < 0.01; **Figure 7A**). In addition, at day 7 following *P. aeruginosa* colonization increased IFN-γ secretion could be detected in MLN of both human and murine fecal microbiota transplanted mice (*p* < 0.001 and *p* < 0.01, respectively; **Figure 7**), whereas IL-10 levels remained unchanged (**Figure 7**). Hence, rather early in the course of murine *P. aeruginosa* colonization intestinal pro-inflammatory, but not anti-inflammatory cytokine responses were induced.

### Systemic Cytokine Responses upon *P. aeruginosa* Colonization of Mice Harboring a Complex Human vs. Murine Gut Microbiota

**FIGURE 8:**
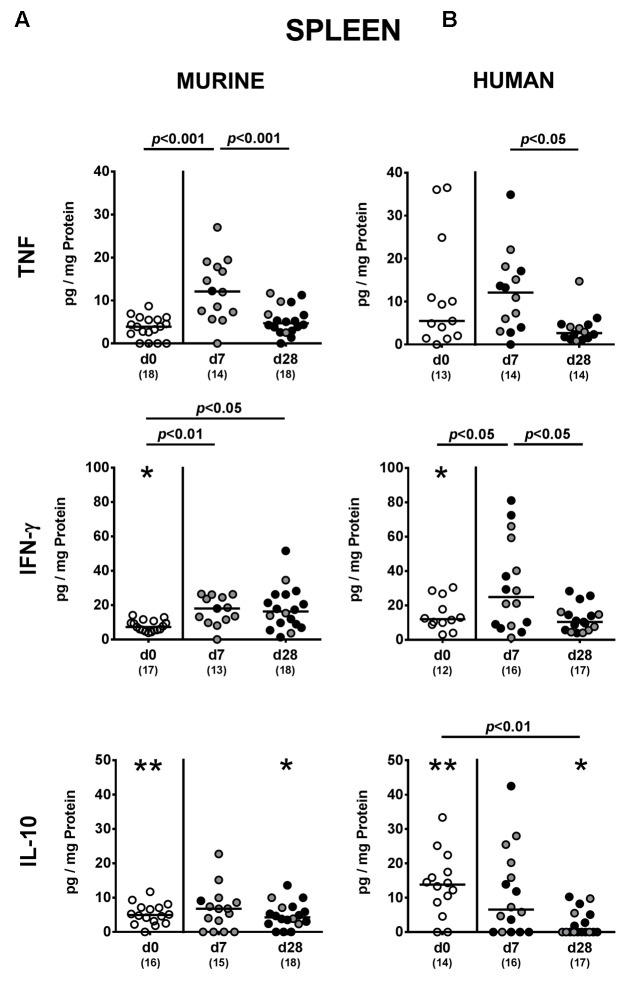
Systemic pro- and anti-inflammatory cytokine responses following MDR *P. aeruginosa* colonization of mice harboring a human vs. murine microbiota. Mice with a **(A)** murine or **(B)** human intestinal microbiota were perorally challenged with MDR *P. aeruginosa* on day 0 and day 1. At day 7 and day 28 post-challenge, TNF, IFN-γ and IL-10 concentrations were determined in splenic *ex vivo* biopsies derived from colonic *P. aeruginosa* carrying (black circles) and non-carrying (gray circles) mice. Unchallenged mice (day 0, open circles) harboring a respective murine or human gut microbiota served as negative controls. Medians (black bars) and significance levels (*p*-values) determined by one-way ANOVA or Kruskal–Wallis test (according to data distribution) are shown. Significant differences between mice with a murine vs. human microbiota at defined time points are indicated by asterisks (^∗^*p* < 0.05; ^∗∗^*p* < 0.01), whereas numbered *p*-values indicate differences between mice harboring the same microbiota. Numbers of analyzed samples are given in parentheses. Data were pooled from four independent experiments.

We finally addressed whether MDR *P. aeruginosa* colonization of human and murine fecal microbiota transplanted mice not only induced local (i.e., intestinal), but also systemic cytokine responses and therefore measured pro- and anti-inflammatory cytokine secretion in splenic *ex vivo* biopsies. Seven days following *P. aeruginosa* challenge, increased TNF concentrations could be detected in spleens derived from mice harboring a murine gut microbiota, but decreased to naive levels until day 28 p.i. (*p* < 0.001; **Figure 8**). In mice that had been subjected to human FMT, a trend towards elevated splenic TNF concentrations could be observed at day 7 p.i. (n.s. due to high standard deviations; *p* < 0.05 vs. day 28 p.i.; **Figure 8B**). Whereas INF-γ concentrations were higher in spleens of mice with a murine microbiota at both day 7 and day 28 p.i. (*p* < 0.01 and *p* < 0.05, respectively; **Figure 8A**), splenic INF-γ levels increased in human fecal microbiota transplanted mice at day 7 p.i. only and reached basal concentrations thereafter (*p* < 0.05; **Figure 8B**). At day 28 p.i., IL-10 was down-regulated in the spleen of mice with human FMT (*p* < 0.01; **Figure 8B**). Hence, cytokine responses upon intestinal colonization with MDR *P. aeruginosa* were not restricted to the intestinal tract, but could also be observed systemically.

## Discussion

In recent years the rising incidences of infections with MDR Gram-negative bacterial including *P. aeruginosa* strains has gained increasing attention not only in the medical field, but also in the general public and global health politics (Livermore, 2009; Oliver et al., 2015). Infection and spread of *P. aeruginosa* have been mainly considered as hospital-associated issues so far, particularly in severely ill patients with antibiotic treatment. *P. aeruginosa* has not been considered part of the commensal microbiota and to date, only a few studies are available addressing the prevalence of *P. aeruginosa* carriage in the healthy population ranging from 0 to 24% (Darrell and Wahba, 1964; Shooter et al., 1966; Sutter and Hurst, 1966; Kessner and Lepper, 1967; Stoodley and Thom, 1970; Bleday et al., 1993; Speert et al., 1993; Bonten et al., 1999; Levy, 2000; Kerckhoffs et al., 2011). However, no data are available whether *P. aeruginosa* carriage, particularly of a MDR strain results in distinct host immune responses.

This prompted us to investigate, whether mice harboring a human vs. murine gut microbiota could be stably colonized by a clinical MDR *P. aeruginosa* strain and whether opportunistic pathogenic carriage was associated with intestinal or even systemic inflammatory host responses. Our present study revealed that the complex intestinal microbiota, irrespective whether of human or murine origin, only partially prevented mice from intestinal *P. aeruginosa* colonization. In fact, approximately 50% of mice harboring a human or murine microbiota were *P. aeruginosa*-negative one week upon supra-physiological challenge with 10^9^ viable bacteria, whereas *P. aeruginosa* could be cultured at relatively low median loads of less than 10^4^ CFU per g feces in up to 78% of mice at day 28 p.i. One might argue that the colonization resistance exerted by the complex intestinal microbiota composition was not fully restored upon broad-spectrum antibiotic pre-treatment and a 3-weeks period following complex bacterial reconstitution by FMT. Our previous infection studies performed at identical timelines, however, revealed that secondary abiotic mice reconstituted with a murine gut microbiota were protected from stable *C. jejuni* colonization and hence, from pathogen-induced pro-inflammatory sequelae (Bereswill et al., 2011). In an earlier study ingested *P. aeruginosa* could be cultured from feces derived from three healthy volunteers up to 6 days following peroral challenge without any clinical sequelae. In line with our results, fecal *P. aeruginosa* counts were lower as compared to those that had been ingested and subsequently declined over time post ingestion (Buck and Cooke, 1969).

One needs to take into consideration, however, that the applied mice harboring a human vs. murine gut microbiota following antibiotic microbiota depletion and subsequent FMT has its limitations – just like any experimental model – and does, hence, only to a certain degree mimic human (patho)physiological conditions. For instance, some members of the human microbiota, particularly obligate anaerobic species might be reduced or even lost upon freezing, thawing and further processing of the fecal donor samples (von Klitzing et al., 2017b) and/or might furthermore not have stably established within the intraluminal ecosystem in some cases as indicated by the observed differences in microbiota compositions of respective donor suspensions and fecal samples derived following FMT. Furthermore, behavioral and environmental factors including housing conditions, diet, genetic background, the distinct anatomic compartment with its inherent intraluminal milieu and respective immunological reportoire all together impact the composition of the complex host-specific gut microbiota (Turnbaugh et al., 2009; Neyrinck et al., 2011; Nguyen et al., 2015; von Klitzing et al., 2017b). Nevertheless, when taking both the strengths and the limitations of the applied experimental model into consideration, with respect to their gut microbiota “humanized” mice might constitute valuable tools in dissecting the interactions between gut commensals, (opportunistic) pathogens and host immunity in health and disease (von Klitzing et al., 2017b). For instance, mice with a human microbiota have been applied to unravel intestinal colonization properties of enteropathogens such as *C. jejuni*, *Salmonella enterica*, and *C. difficile* (Bereswill et al., 2011; Chung et al., 2012; Collins et al., 2015).

In the present study mice were also clinically uncompromised during the entire observation period following peroral *P. aeruginosa* challenge. On microscopic level, however, *P. aeruginosa* induced inflammatory sequelae could be assessed as indicated by increased apoptotic epithelial cell numbers in both small and large intestines as early as 7 days p.i., irrespective of the intestinal microbiota composition of mice. Of note, apoptosis was paralleled by increased proliferative/regenerative epithelial cell responses thereby counteracting potential cell damage. Furthermore, *P. aeruginosa* colonization was accompanied by a rather early innate immune response as indicated by increased numbers of macrophages and monocytes in the colonic mucosa and lamina propria of human fecal microbiota transplanted mice, whereas small and large intestinal numbers of adaptive immune cells such as B lymphocytes were elevated at the end of the observation period. Pro-inflammatory sequelae of *P. aeruginosa* colonization are further characterized by increased TNF secretion in both colon and MLN of mice at day 7 p.i., whereas anti-inflammatory IL-10 levels, however, remained unaffected in the intestinal tract. The *P. aeruginosa* induced immune responses are not surprising given the plethora of virulence factors of the opportunistic pathogen. For instance, *Pseudomonas* lipid A, a core component of bacterial lipopeptide, is capable of activating NFκB signaling via TLR-4 leading to pro-inflammatory cytokine secretion (Korneev et al., 2015). Subsequently, innate immune cells are recruited to the site of infection contributing to inflammatory host responses directed against *P. aeruginosa* (Gellatly and Hancock, 2013).

Strikingly, immune responses upon *P. aeruginosa* carriage were not restricted to the intestinal tract, but could also be observed systemically, given that TNF as well as IFN-γ concentrations were elevated in spleens as early as 7 days p.i., whereas splenic IL-10 levels were dampened in human fecal microbiota transplanted mice later-on. To the best of our knowledge, we here for the first time show that mere intestinal carriage of MDR *P. aeruginosa* by a clinically unaffected host results in pronounced intestinal as well as systemic sequelae. This is even more surprising, given that not all mice under investigation were stable *P. aeruginosa* carriers further underlining the pathogenic potential of MDR *P. aeruginosa*.

Despite several reports of diarrhea caused by *P. aeruginosa* in patients with prior antibiotic treatment, *P. aeruginosa* is not regarded as a common intestinal pathogen in the healthy host (Adlard et al., 1998; Kim et al., 2001). As early as 1918, however, “Shanghai fever”, a community acquired syndrome characterized by diarrhea, fever and *P. aeruginosa* sepsis with high mortality has been reported affecting neonates and children without pre-existing comorbidities primarily in Asian populations (Dold, 1918; Chusid and Hillmann, 1987; Martin-Ancel et al., 1993; Viola et al., 2006; Chuang et al., 2014).

Given the potential cross-talk between (opportunistic) pathogens and commensal intestinal bacteria, we addressed whether changes in respective gut microbiota compositions might occur during *P. aeruginosa* colonization. Interestingly, no overt gut microbiota shifts could be observed early (i.e., 1 week) upon *P. aeruginosa* colonization and were only minor in human fecal microbiota transplanted mice thereafter. Previous studies revealed that both acute and chronic small as well as large intestinal inflammation in mice were associated with increased luminal loads of commensal EB further perpetuating the inflammatory scenario via TLR-4 dependent signaling of lipopolysaccharide (LPS) as Gram-negative bacterial cell wall constituent (Heimesaat et al., 2006, 2007a,b, 2010; Erridge et al., 2010; Haag et al., 2012a,b; Otto et al., 2012). However, despite observed pro-inflammatory immune responses with pronounced intestinal epithelial apoptosis, sequelae of intestinal MDR *P. aeruginosa* carriage was not sufficient enough to drive gut microbiota shifts towards overgrowth with potentially pro-inflammatory commensals.

## Conclusion

Our study demonstrates that in with a complex microbiota reconstituted secondary abiotic mice the established intestinal microbiota (irrespective whether of human or murine origin) does not sufficiently prevent the host from intestinal colonization with a MDR *P. aeruginosa* strain. Furthermore, mere intestinal carriage of MDR *P. aeruginosa* pose otherwise healthy hosts at risk of developing not only intestinal but even systemic pro-inflammatory sequelae. Future studies need to further unravel the molecular mechanisms underlying the interactions between *P. aeruginosa*, the commensal microbiota and host immunity in health and disease in order to develop strategies preventing from colonization with MDR Gram-negative strains including *P. aeruginosa*.

## Author Contributions

EvK: designed and performed experiments, analyzed data, co-edited paper. IE: performed experiments, analyzed data, co-edited paper. SB: provided advice in design and performance of experiments, co-edited paper. MH: designed and performed experiments, analyzed data, wrote paper.

## Conflict of Interest Statement

The authors declare that the research was conducted in the absence of any commercial or financial relationships that could be construed as a potential conflict of interest.

## Supplementary Material

FIGURE S1 Fecal MDR *P. aeruginosa* loads in mice harboring a human vs. murine microbiota. Mice with a **(A)** murine or **(B)** human intestinal microbiota were perorally challenged with MDR *P. aeruginosa* on day 0 and day 1. Intestinal colonization densities were assessed in fecal samples at defined time points p.i. by culture. Medians (black bars) and significance levels (*p*-values) determined by Kruskal–Wallis test are shown. Numbers of samples harboring the respective bacterial group out of the total number of analyzed samples are given in parentheses. Data were pooled from four independent experiments.

FIGURE S2 Apoptotic and proliferating epithelial cell as well as immune cell responses in the small intestines following MDR *P. aeruginosa* colonization of mice harboring a human vs. murine microbiota. Mice with a **(A)** murine or **(B)** human intestinal microbiota were perorally challenged with MDR *P. aeruginosa* on day 0 and day 1. At day 7 and day 28 post-challenge, the average number of apoptotic (Casp3+) and proliferating (Ki67+) cells as well as of macrophages/monocytes (F4/80+) and B lymphocytes (B220+) in at least six HPF were quantitatively assessed in ileal paraffin sections derived from colonic *P. aeruginosa* carrying (black circles) and non-carrying (gray circles) mice applying *in situ* immunohistochemistry. Unchallenged mice (day 0, open circles) harboring a respective murine or human gut microbiota served as negative controls. Medians (black bars) and significance levels (*p*-values) determined by Mann–Whitney *U*-test are shown. Significant differences between mice with a murine vs. human microbiota at defined time points are indicated by asterisks (^∗^*p* < 0.05; ^∗∗^*p* < 0.01; ^∗∗∗^*p* < 0.001), whereas numbered *p*-values indicate differences between mice harboring the same microbiota. Numbers of analyzed samples are given in parentheses. Data were pooled from four independent experiments.

## Abbreviations

Bif: bifidobacteria
B/P: *Bacteroides/Prevotella* spp.
CFU: colony forming units
*C. jejuni*: *Campylobacter jejuni*
Clept: *Clostridium leptum* group
Clocc: *Clostridium coccoides* group
EB: enterobacteria
EC: enterococci
FMT: fecal microbiota transplantation
HPF: high power fields
ICU: intensive care unit
LB: lactic acid bacteria
MDR: multi-drug resistant
MIB: *Mouse Intestinal Bacteroides*
MLN: mesenteric lymph nodes
*P*: two-sided probability values
PBS: phosphate buffered saline
p.i.: post-infection
qRT-PCR: quantitative real-time polymerase chain reaction
SPF: specific pathogen-free
TL: total eubacterial load
TLR: Toll-like receptor
WHO: World Health Organization
