# Supplementary figures and images for: Intestinal and Systemic Immune Responses upon Multi-drug Resistant Pseudomonas aeruginosa Colonization of Mice Harboring a Human Gut Microbiota
Source: Front Microbiol. 2017 Dec 22;8:2590. doi: 10.3389/fmicb.2017.02590 (PMC5744425; doi:10.3389/fmicb.2017.02590)

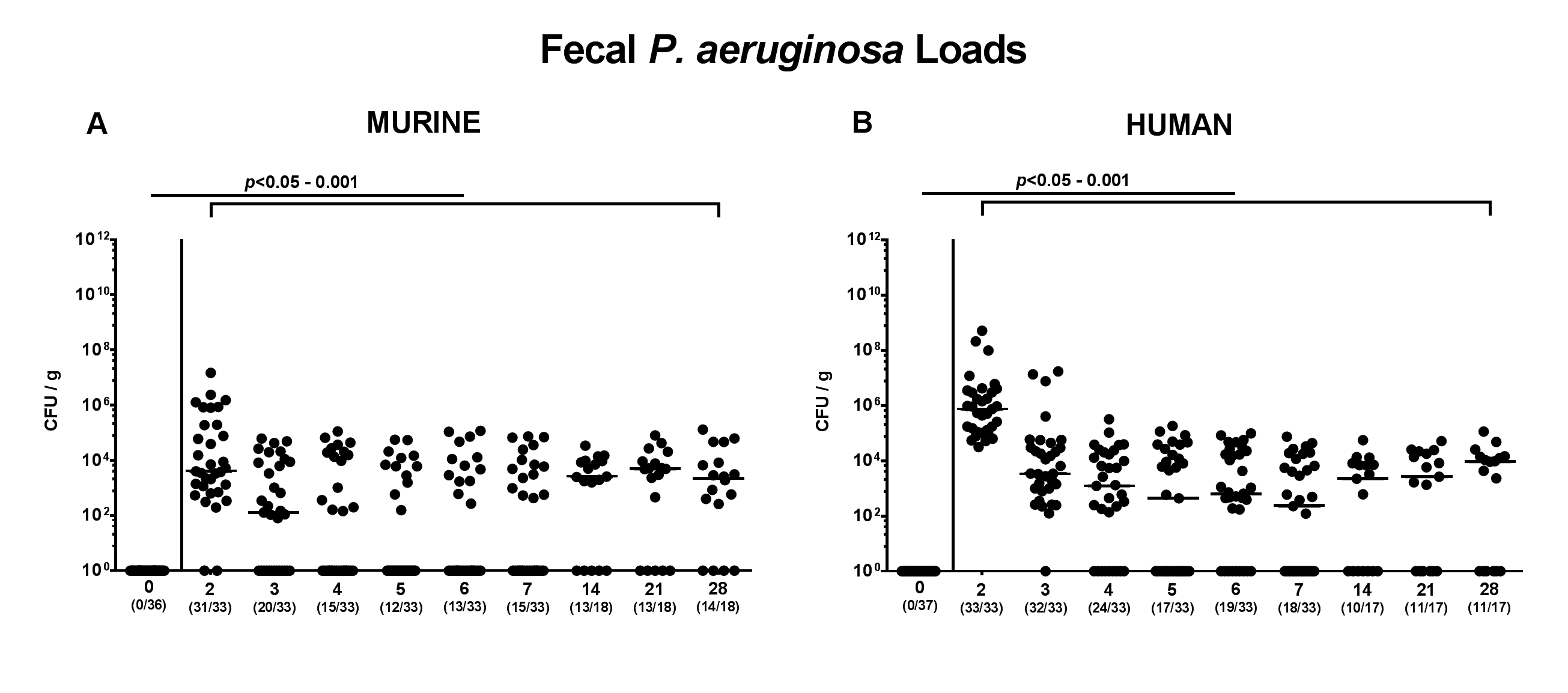

Supplement: FIGURE S1 — Fecal MDR P. aeruginosa loads in mice harboring a human vs. murine microbiota. Mice with a (A) murine or (B) human intestinal microbiota were perorally challenged with MDR P. aeruginosa on day 0 and day 1. Intestinal colonization densities were assessed in fecal samples at defined time points p.i. by culture. Medians (black bars) and significance levels (p-values) determined by Kruskal–Wallis test are shown. Numbers of samples harboring the respective bacterial group out of the total number of analyzed samples are given in parentheses. Data were pooled from four independent experiments. [file Image_1.TIFF]

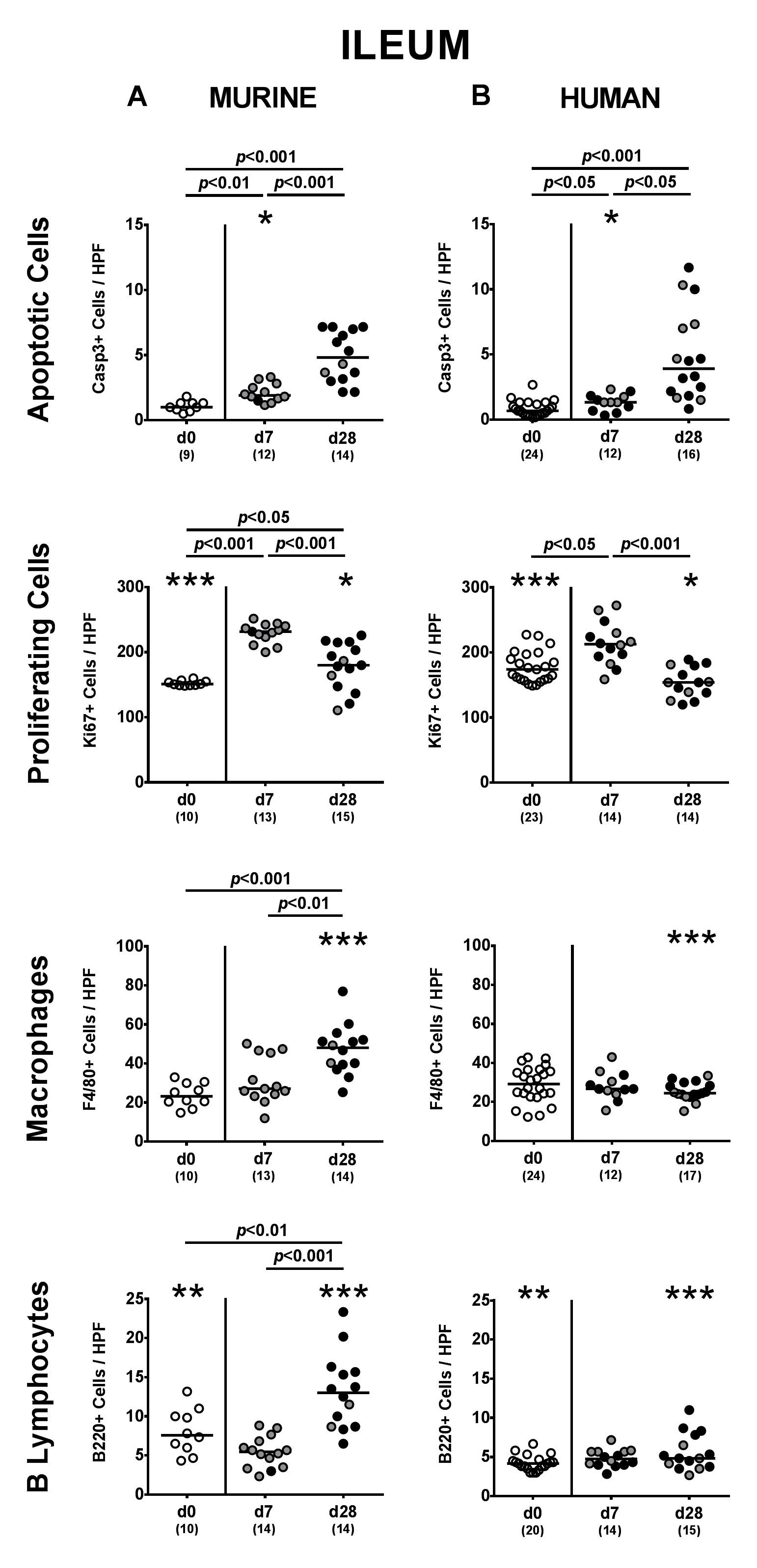

Supplement: FIGURE S2 — Apoptotic and proliferating epithelial cell as well as immune cell responses in the small intestines following MDR P. aeruginosa colonization of mice harboring a human vs. murine microbiota. Mice with a (A) murine or (B) human intestinal microbiota were perorally challenged with MDR P. aeruginosa on day 0 and day 1. At day 7 and day 28 post-challenge, the average number of apoptotic (Casp3+) and proliferating (Ki67+) cells as well as of macrophages/monocytes (F4/80+) and B lymphocytes (B220+) in at least six HPF were quantitatively assessed in ileal paraffin sections derived from colonic P. aeruginosa carrying (black circles) and non-carrying (gray circles) mice applying in situ immunohistochemistry. Unchallenged mice (day 0, open circles) harboring a respective murine or human gut microbiota served as negative controls. Medians (black bars) and significance levels (p-values) determined by Mann–Whitney U-test are shown. Significant differences between mice with a murine vs. human microbiota at defined time points are indicated by asterisks (∗p < 0.05; ∗∗p < 0.01; ∗∗∗p < 0.001), whereas numbered p-values indicate differences between mice harboring the same microbiota. Numbers of analyzed samples are given in parentheses. Data were pooled from four independent experiments. [file Image_2.TIFF]
